# The Paradox of Social Interaction: Shared Intentionality, We-Reasoning, and Virtual Bargaining

**DOI:** 10.1037/rev0000343

**Published:** 2022-04

**Authors:** Nick Chater, Hossam Zeitoun, Tigran Melkonyan

**Affiliations:** 1 Warwick Business School, University of Warwick; 2 Department of Economics, Finance and Legal Studies, University of Alabama

**Keywords:** shared intentionality, coordination, social interaction, we-reasoning, mind-reading

## Abstract

Social interaction is both ubiquitous and central to understanding human behavior. Such interactions depend, we argue, on shared intentionality: the parties must form a common understanding of an ambiguous interaction (e.g., one person giving a present to another requires that both parties appreciate that a voluntary transfer of ownership is intended). Yet how can shared intentionality arise? Many well-known accounts of social cognition, including those involving “mind-reading,” typically fall into circularity and/or regress. For example, *A*’s beliefs and behavior may depend on her prediction of *B*’s beliefs and behavior, but *B*’s beliefs and behavior depend in turn on her prediction of *A*’s beliefs and behavior. One possibility is to embrace circularity and take shared intentionality as imposing consistency conditions on beliefs and behavior, but typically there are many possible solutions and no clear criteria for choosing between them. We argue that addressing these challenges requires some form of we-reasoning, but that this raises the puzzle of how the collective agent (the “we”) arises from the individual agents. This puzzle can be solved by proposing that the will of the collective agent arises from a simulated process of bargaining: agents must infer what they *would* agree, were they able to communicate. This model explains how, and which, shared intentions are formed. We also propose that such “virtual bargaining” may be fundamental to understanding social interactions.

We intuitively explain human behavior, including our own, by giving reasons that may justify that behavior (Davidson, 1963; Dennett, 1987). Our reason-based explanations of each other’s actions are often multilayered and complex. So, for example, when buying a used car in cash, we may explain why *A* carefully counts the money and holds it up to the light by her suspicion that *B* is dishonest. We may further explain that *B* feels affronted because *A* is demonstrating a lack of trust. Going a step further, we can explain why *A* remarks, “I suppose I’ve got to go through this rigmarole!” to reduce *B*’s sense of being distrusted, by implying that *A* makes such checks as a matter of routine.^1^

Many perspectives on social behavior elaborate on these intuitive, reason-based explanations of individual thought and behavior (e.g., Ajzen, 1991; Bandura, 1982; Fishbein, 1979; Mercier & Sperber, 2011; Perner, 1991). One direction for such elaboration, rooted in cognitive science, focuses on the rich structures of prior knowledge and experience that provide flexible “scripts” for common types of interaction (Minsky, 1974; Schank & Abelson, 1977). A second focuses on the complex role-playing and turn-taking conversational structure of many social interactions (e.g., Goffman, 1959; Haviland & Clark, 1974; Levinson & Torreira, 2015). A third focuses on the pragmatic inferences required to infer the meaning of linguistic and nonlinguistic social cues (Clark, 1996; Grice, 1975; Sperber & Wilson, 1986). These and other projects go far beyond common-sense by extending and deepening, rather than replacing, reason-based explanations of social behavior.

Psychology as a discipline focuses primarily on the individual, and on the mental processes of which individual thoughts and behaviors are composed. Thus, psychological explanation typically, as in the example above, attributes reasons to individual people. From this point of view, organizations, social classes, and nations are not irreducibly ascribed “motives” or “beliefs.” Instead, any idea of reason or agency of a group should be explained purely in terms of the motives and beliefs of its members. It has generally been left to other social sciences, such as sociology, economics, management, and political science, to explain how group-level agency can arise from interactions between the individual human agents of which they are composed.

Recently, though, psychologists and philosophers have become increasingly interested in reason-based explanations that apply to more than one person, as embodied in notions of joint action, joint attention, and shared intentionality. We shall use “shared intentionality” broadly, to cover any cases in which people reach a commonly agreed interpretation, understanding, decision, or plan.^2^ Shared intentionality—including the ability to act, attend, and plan jointly—has been viewed as a key feature distinguishing humans from other primates (e.g., Call, 2009; Tomasello & Rakoczy, 2003). Moreover, shared intentionality has been seen as crucial to the development of culture (Searle, 1995; Tomasello et al., 2005); as playing a key role in cognitive and social development (Tomasello & Carpenter, 2007); supporting coordinated physical activities (Sebanz et al., 2006); shaping and directing individuals’ attentional and emotional resources (Boothby et al., 2014; Shteynberg & Apfelbaum, 2013; Shteynberg et al., 2014); motivating people to coordinate their efforts (Thomas et al., 2014); and providing a foundation for language (Clark, 1996; Clark & Brennan, 1991; Tomasello, 2008). Indeed, as we shall see below, social interactions of all kinds may often involve shared intentionality as a key element.

In philosophy and economics, the question of how or whether shared intentionality can be explained in terms of the thoughts and behaviors of individual agents has been viewed as a major theoretical challenge (e.g., Bacharach et al., 2006; Bratman, 1992, 1993; Gilbert, 2006; Searle, 1990). By contrast, in psychology, we suggest that the size of the gulf between reason-based explanation of single individuals and reason-based explanation of the *interactions* between individuals is often not fully appreciated.^3^ Indeed, it is often assumed that the ability to “read” the minds of others may be sufficient, typically with the addition of so-called “higher order” intentionality, recognizing that the others are also mind-readers. In this paper, we will argue that this is not the case.

The problem of “shared,” or “joint,” reasoning to reach some common understanding about each other’s thoughts and behavior is, as we shall see, widespread. But it has clear boundaries: not all interactions between people have a fundamentally collaborative character aiming to establish a common understanding or plan. Consider, for example, situations in which one individual interacts with another purely as a physical “object,” whether deliberately pushing someone out of the way, or inadvertently knocking them over. Equally, many aspects of social interaction are fundamentally one-directional: one party is attempting to interpret the behavior of another, ascribing them beliefs and motivations; or one person is attempting to control or manipulate the thoughts or behavior of another (e.g., so-called Machiavellian intelligence, widely discussed in primatology, e.g., Byrne & Whiten, 1989; de Waal, 2007). Indeed, much social behavior is fundamentally competiti*v*e, so that each party aims to understand and/or control the behavior of the other to their own advantage. In highly competitive interactions, the goal is typically not to reach a common understanding, but to outwit the other. Moreover, even where a common understanding is crucial to successful interaction, this need not be obtained by joint reasoning between the parties involved. A common understanding of the rules of the road, or the rules of tennis, or chess, or indeed linguistic conventions and moral norms, need not be created from scratch in interacting with another person, but may be presupposed as part of a shared culture.^4^

Yet collaborative, shared reasoning can often play a crucial role in even these cases. Although pushing another person does not involve collaborative, shared thinking, the evaluation of such actions (e.g., as impolite), and whether apology is required, or protest justified, may well do. If *A* pushes *B* out of the path of an oncoming vehicle that *B* had not noticed, there may be a joint expectation that *A* will be thanked rather than reprimanded. Similarly, in playing tennis, coordinating on when and where to play, or what is appropriate in a warm up involves shared intentionality; but in a competitive rally, each player will aim to disguise her intentions from, rather than share her intentions with, the other. Moreover, behavior is often guided by the interpretation of the rules (e.g., deciding whether a soccer shot is “over the bar” or “hits the post” where the goal is marked by two jumpers on a children’s playground). Forming such tacit agreements seems paradigmatically to involve shared intentionality (e.g., Searle, 1995). Moreover, finding such shared interpretations will be particularly important in verbal communication in the light of the highly underspecified nature of linguistic meanings (Grice, 1975; Levinson, 2000; Sperber & Wilson, 1986; Turner & Horn, 2018) and the need to “anchor” those meanings in a specific context of people, objects, locations, and so on (Clark, 2021). But, once the rules are commonly established, playing the game itself is not a matter of shared intentionality. In chess, for example, the action of “launching an attack on the King” does not depend on mutual recognition by both players: indeed, the attack is more likely to succeed if the other fails to recognize it.

Shared intentionality arises where people need to share the interpretations of relevant actions, but it is puzzling how such a shared interpretation can arise. Specifically, attempting to reach such a shared interpretation runs into what we call the problem of *mutual prediction*. For person *A* to find a shared understanding of an action with *B*, person *A* needs to infer *B*’s understanding of that action. But *B*’s understanding will, in turn, be shaped by *B*’s attempt to infer *A*’s understanding. Thus, *A*’s thoughts and actions depend on *B*’s; and in turn *B*’s thoughts and actions depend on *A*’s in an apparent loop.^5^

As we will see, this seemingly innocuous observation is surprisingly problematic, and indeed paradoxical, for reason-based accounts of social interaction involving the formation of shared intentions. We consider various attempts to address this challenge and suggest a solution using the theory of virtual bargaining (Melkonyan et al., 2018, 2021; Misyak & Chater, 2014; Misyak et al., 2014).

## Outline of the Paradox

Let us introduce the paradox with a simple example. *A* passes *B* a copy of *A*’s latest book. *B* has to determine the nature of this social interaction from ambiguous clues. Let us limit ourselves to two possibilities: that *A* may be *giving* (*G*) the book to *B*, in which case *B* should thank *A* profusely and keep the book; or *A* may be *showing* (*S*) the book to *B*, in which case *B* should examine it with interest, make suitably obliging remarks, and return it. A shared interpretation is crucial for harmonious social interaction. In the language of game theory (Schelling, 1960), social interactions require that *A* and *B* play a *coordination* game in order to resolve this ambiguity between *G* and *S*.^6^

At first glance, the challenge of making the correct inference may seem to lie entirely with *B*. That is, *B* has to engage in some process of mind-reading to establish *A*’s intention (*G* or *S*?). But note that *A* faces the complementary problem: predicting how *B* will interpret being handed the book.

For both parties, a successful communicative and social interchange requires that their interpretations, whether *G* or *S*, are the *same*. Indeed, considerable social confusion and embarrassment will result if *A* believes the book to be a generous gift, yet *B* hands the book back after a brief look and a few perfunctory remarks. Matters may be even worse if *A* intended *B* merely to inspect the book, but *B* gushes with thanks about *A*’s kindness and stubbornly fails to release it.

The two parties face a problem of mutual prediction. In rationally deciding on how or whether to pass the book to *B*, *A* must ask: will *B* interpret my action as *G* or *S*? But to decide this, *A* must ask what *B* will think *A* intends; and *B* will, of course, assume that *A* intends whatever *B* will infer from observing *P*. *A* and *B* seem to be reasoning in a circle: *A* is trying to “read” *B*’s mind; but *B* is trying to read *A*’s.

Thus, mutual prediction seems to lead inexorably to deadlock or infinite regress. We call this the *paradox of social interaction*. The paradox seems to arise widely. In many social interactions, there will be the mutual challenge of inferring what the nature of the interaction is from ambiguous information: for example, who is supposed to play which role and take which actions (e.g., Clark, 1996; Goffman, 1959). We stress that the paradox of social interaction is a challenge for theorists, rather than individuals. Specifically, the puzzle for theorists is how, given the apparent threat of deadlock or regress, people are able to navigate reciprocal interactions with other people so deftly.

Three points are worth stressing. First, any specific ambiguity in a social situation may, of course, be resolved, or at least reduced, by one or both parties. The person passing the book might present it ostentatiously, in order to clarify that it is a gift; or strongly imply that it is not (e.g., by commenting “you can have a look, if you like”). Nonetheless, most social situations will retain *some* measure of ambiguity. For example, even if it is made clear that the book is presented for inspection, there remains uncertainty about, for example, how rapidly it should be returned, how delicately it should be handled, or whether or not permission is required to scan its cover or opening pages with a smartphone. And explicit communication, however, extensive, never resolves all ambiguities—this is parallel with the observation in legal theory that the law is inevitably “open-textured,” and open to further interpretation (Hart, 1994). Indeed, natural language itself is notoriously riddled with ambiguities, including the scope of quantifiers, the extensions of concepts, the reference of names and pronouns, and so on (Sennet, 2016). Thus, the challenge of coordinating on a shared understanding through coordination is not eliminated by the possibility of communication, but rather recurs in the interpretation of communication.

Second, disambiguation is itself a subtle and sensitive social action. *A*’s overzealous signaling that the book should be returned will imply a concern that *B* would otherwise be likely to keep it. *A*’s signaling may impugn *B*’s level of social competence by implying that *B* is liable to make social gaffes; or impugn *B*’s motives by implying that *B* might be deliberately exploiting a potentially ambiguous situation to make off with the book. For such reasons, people will typically exercise caution in explicating what otherwise might reasonably be inferred to be the shared understanding by both parties.

Thirdly, note that agreeing on the nature of the social interaction seems to be required even where the interaction may be hostile. For example, even an overtly angry act is not thereby necessarily *hostile*. *B* might furiously tear up *A*’s book without concern for whether *A* is observing or even present. *B*’s act is hostile only when it is expressly aimed at conveying *A* and *B*’s shared recognition of *B*’s contempt for the book (and perhaps also for its author). Or suppose *B*, avenging some past slight, attempts to insult *A* by putting the book into the trash. *B*’s intention is that both *A* and *B* will agree that this action is an insult to the book’s author, *A*. If *A* misinterprets *B*’s behavior as accidental, for example, it will not have the desired insulting effect.

What makes putting the book into the trash part of a hostile *interaction* is that *A* and *B* share the interpretation of the action as a deliberate insult. Thus, *B* is actively attempting to insult *A*, and *B*’s success requires that *A* recognizes this intention; that *B* recognizes that *A* has this recognition; and so on. In the view of the psychology of language, this understanding of the action as an insult is in *common ground* (Clark, 1996). While common ground is a subtle notion, and one which we will use further below, we need only an informal understanding of the idea here: for some piece of information to be in common ground for *A* and *B*, we require that they both know it; know that they each other know it, and so forth.^7^ From a mutual prediction perspective, it is hard to see how a common ground interpretation of *B* putting *A*’s book in the trash can ever arise. *A* and *B* seem to be led down an endless regress in which each tries to infer how the other would interpret the action.

Social interactions, then, frequently involve generating shared intentions and shared understanding from potentially ambiguous cues. This, in turn, seems to generate an apparently paradoxical interdependence between *A* and *B*: the thoughts and actions of each seem to depend on the thoughts and actions of the other. How, then, might we attempt to escape the paradox, and deal with the mutual interdependence of *A* and *B*’s actions?

One approach is to embrace circularity: to note simply that there are two “readings” of the action which are consistent and, if adopted, will lead to harmonious interaction. On one reading, *A* and *B* both interpret the physical movements of conveying the book from one person to the other as an instance of *S*; both believe that the other adopts reading *S*, that the other believes that the other believes that they adopt reading *S*, and so on. On the other reading, both interpret the same physical movements as a case of *G*, with the parallel hierarchy of beliefs, and beliefs about beliefs. But the paradox remains: how do the parties know *which* interpretation to agree on?

It is tempting to suppose that solving coordination problems is, in practice, often made easier by a clue or hint. Indeed, *A* might wave the book toward *B* very casually, rather than slowly and formally, perhaps seeking to imply, “this is no big deal—just have a quick look,” and this might seem to imply that *A* does not intend to give the book as a gift. But notice that any hint will work only if both parties *agree* on the interpretation of the hint (e.g., that it favors *S* rather than *G*). But then both parties face a new coordination problem: coordinating on the interpretation of the “hint,” and the paradox is just as problematic as before.

### Why Has the Paradox Been Largely Unnoticed in Psychology?

We have suggested that the paradox of social interaction is fundamental. But discussion of this, and related, issues is sparse in psychology. There are notable exceptions, including analysis of these issues in the context of language and communication (e.g., Clark, 1996), and in social (Shteynberg et al., 2020) and developmental (Bohn & Koymen, 2018) psychology. Moreover, there is work showing the power of common ground in experimental games (Colman, 2003; Colman & Gold, 2018; De Freitas et al., 2019; Thomas et al., 2014); and proposals concerning the need for evolved cognitive mechanisms to think “as a group” (Tooby & Cosmides, 2010). But given that social interaction is so central to human life, it is natural to ask why the paradox has not been a major focus of psychological discussion.

One reason is, we suspect, that humans are so good at resolving such coordination problems in practice that their very existence is not noticed: the “right” interpretation of an action (e.g., a gift, an insult) seems so obvious that it may seem to require no further explanation. Another reason is that a great deal of experimental research, even in social psychology, does not involve *interaction*: indeed, for good reasons of experimental control, paradigms involving the interplay of freely interacting agents are often avoided where possible.^8^

In short, social interaction is often not the immediate focus of investigation in social psychology and related fields. For example, considerable interest has focused on what we might term *social perception* (e.g., Heider, 1958; Kelley, 1967; Macrae & Bodenhausen, 2000) or *mind-reading* (Baron-Cohen et al., 2001; Nichols & Stich, 2003; Singer, 2006). Here people infer another person’s mental state from their observed behavior, but where the person observed is not simultaneously making an inference about the observer. Similarly, there is considerable interest in how a person’s thoughts and behavior are affected by people and groups around them. *Social influence* (e.g., Asch, 1956; Cialdini & Goldstein, 2004; Sherif, 1936) too, need not be reciprocal (although it can be: Mahmoodi et al., 2018). A person’s thoughts and behaviors will be shaped by those around them, irrespective of whether the converse is true. Another area of intensive research interest is the *social transmission* of thoughts and behaviors through populations and across generations (e.g., Boyd et al., 2011; Christakis & Fowler, 2009; Heath et al., 2001). The spread of linguistic expressions, gestures, motor skills, levels of aggression, food and alcohol consumption, norms of politeness, emotions, rumors, and beliefs is typically considered as involving one-way transfer from one agent to another. In each of these important domains, the relationship between one agent and the next can be modeled as one-directional, without needing to invoke mutual prediction. Table 1 provides an illustrative sample of well-known experimental studies in social, developmental, and comparative psychology, under this classification.[Table tbl1]

It is perhaps natural to hope that a theory of social interaction might be created by adding a one-directional influence from *A* to *B* to a one-directional influence from *B* to *A*. But the paradox of social interaction illustrates how mutual influence can introduce wholly new phenomena: people must simultaneously and independently infer relevant beliefs, intentions, and actions for both parties.^9^ To the degree that researchers have focused on one-way interactions and anticipated a natural generalization to the two-way case, the paradox of social interaction has been largely unnoticed.

### The Paradox and Some Current Theoretical Accounts

Many influential reason-based theories of social development, cognition, and behavior appear either (a) to run into the paradox or (b) to leave it unresolved. In the first category are process models that, implicitly or otherwise, propose that person *A* attempts to mind-read person *B*, taking account of the fact that *B* is attempting to mind-read person *A*, and so forth. In the second category are models that embrace circularity by defining “equilibrium conditions” under which *A*’s and *B*’s beliefs and/or actions can be consistent. But, as we noted above, this side-steps the crucial question—how do *A* and *B* coordinate on *which* of the possible multiple equilibria to choose (do they both see the handing over of the book as giving or showing?).

The first problem (circularity) applies to prediction-based models, which are widespread in the cognitive and brain sciences (Clark, 2013), and have recently been applied to social behavior (e.g., Tamir & Thornton, 2018). According to this viewpoint, the brain is a “prediction machine” with a fundamental drive to learn by reducing its prediction error. But in the context of mutually interdependent social interaction, this approach treats social interaction as a problem of mutual prediction, and our paradox hits with full force. If *A* and *B* are two “prediction machines” each attempting to predict the other, they are seemingly unable to avoid an infinite regress.

The same issue recurs, independent of the mechanism used to make such predictions. For example, consider the simulation-theory of mind (e.g., Goldman, 2006; Gordon, 1986), according to which people attempt to mind-read by simulating the thinking of others using their own knowledge and reasoning. The essential claim of the simulation-theory is that, rather than requiring a “theory” or “model” of the mind of the other, the other’s mind can be predicted by using one’s own mind as an analog of the other’s mind. Consider, for example, a criminal wondering how to mislead a detective by leaving false “clues.” According to the simulation theory, to assess what a detective will infer from seeing a clue, the criminal asks herself what she herself would infer from seeing that same clue. This might involve modifying her own beliefs appropriately to align with those of the detective. Thus, the criminal might ask what, hypothetically, she would infer from a clue, were she not already aware of her own guilt; or what she would infer if, like the detective, she were convinced that no crime could be committed by a judge or a member of the clergy. Whatever the attractions of this account, it has limited purchase on the paradox of social interaction. In such situations, each person is attempting to simulate the other. Thus, *A* needs to simulate *B* simulating *A* simulating *B*, and it is not clear how infinite regress can be avoided. The simulation theory just embodies the paradox, rather than helping to resolve it.

The picture is no different for the influential theory-theory of mind (Gopnik & Wellman, 1992; Wellman, 1992), according to which people infer the mental states of others to best explain their behavior. That is, an observer builds a “model” of the thoughts of the other. This approach works well for social perception, where one person observes, but does not interact with, another person. Indeed, this viewpoint has been productively modelled by extending Bayesian methods that have been so successful in capturing the perception of the physical world (e.g., Knill & Richards, 1996; Tenenbaum et al., 2011; Yuille & Kersten, 2006) and social behavior (Baker, 2012; Baker et al., 2009, 2017).

In social interaction, though, this recursive approach runs into a version of our now-familiar paradox. Consider *A* and *B*’s interaction above. According to the theory-theory viewpoint, to understand *B*, *A* must have a model of *B*’s reasoning. But conversely, to understand *A*, *B* must have a model of *A*’s reasoning. This seems to imply that *A*’s model of *B*’s reasoning must contain a nested model of *A*’s reasoning, which presumably in turn will include a further model of *B*’s reasoning—we have our usual problem of infinite regress. Most psychological accounts of this type implicitly or explicitly assume that such recursion is truncated (e.g., Tomasello, 2008). That is, people model one another’s beliefs (“first-order” beliefs); perhaps also model that others model their own beliefs (“second-order” beliefs); and perhaps occasionally extend to the third and fourth order (Liddle & Nettle, 2006).

A variant of this approach is also embodied in formal theories in behavioral game theory (cognitive hierarchy theory; Camerer et al., 2004; and level-k reasoning; Costa-Gomes & Crawford, 2006; Nagel, 1995; Stahl & Wilson, 1994). This truncated reasoning approach, while helpful in analyzing certain kinds of competitive interactions, has difficulties in dealing with coordination. Consider, for concreteness, the cases where *A* and *B* are both second-order reasoners. *A* then tries to do whatever she thinks *B* thinks she (*A*) will do.^10^ But knowing what *B* thinks *A* will do is at least as difficult as deciding what *A* will do herself. And *B* faces a similar challenge, trying to establish what *A* thinks he will do, when he has yet to decide this himself. Of course, agents might use some heuristic to infer these difficult second-order beliefs. But, if so, then (a) the entire explanatory burden falls on the heuristics, not the theory of mind-reading; (b) if such heuristics existed, both parties should surely apply them directly to determine their own behavior and might avoid becoming lost in unnecessary recursive reasoning; and (c) the question of how the heuristics emerge to lead to successful coordination is entirely unaddressed. In sum, theories that assume that people’s recursive mind reading is limited to a finite number of levels cannot easily explain how people coordinate successfully in social interactions.^11^

The second category of models, as we have noted, embraces circularity and seeks to define a notion of equilibrium, in which the actions and/or beliefs of the parties are consistent (although then facing the puzzle of how people are able to coordinate by choosing the same equilibrium). This approach is standard in game theory in the form of the Nash equilibrium and its many variants. It is also embodied in some sophisticated Bayesian computational models of social reasoning in psychology. For example, consider the social interaction of “optimal” teaching in which an agent aims to present the information that will be most useful to a learner. Shafto et al. (2014) define Bayesian equations in which teachers (approximately) optimize their choice of an action in the light of their model of the listener, and listeners optimize their interpretations of the information from the teacher, based on their model of the teacher; and use iterations to enhance their consistency. This approach has been extended to deal with pragmatic reasoning, with the creation of rational speech act theory (Frank & Goodman, 2012; Goodman & Frank, 2016). This work is interestingly related to the notion of “rationalizability” in game theory (Bernheim, 1984; Pearce, 1984), in which players’ choices can be rationalized as a best response in the light of some beliefs about the other players (though these beliefs need not be correct). As Shafto et al. (2014) noted, their approach, like rationalizability, typically generates many possible solutions. Therefore, some additional mechanism is required to select a specific prediction.

One particularly interesting approach along these lines, which can be viewed as formalizing the theory-theory approach, is “action interpretation as inverse planning” (Baker et al., 2009; Wang et al., 2021). The aim is to provide a reason-based account of social perception by applying a Bayesian analysis-by-synthesis approach, widely used in modeling perception (Yuille & Kersten, 2006) and language interpretation (Bever & Poeppel, 2010), to the problem of understanding the intentions behind observed actions. The approach begins with prior assumptions about the rational planning of actions in the light of goals and beliefs and uses Bayesian inference to invert this model and hypothesize goals, beliefs, and plans from the observed action. This work provides an elegant account of how it is possible to “read” complex beliefs and preferences from behavior. For example, if we see an agent circumspectly skirt an area of ground while heading toward a goal, we may tentatively infer that the agent believes there is some hidden obstacle or danger that would be encountered on the direct route—otherwise their behavior would not correspond to an optimal plan.

Like other approaches to social perception, this strategy would lead to deadlock if directly applied to social interactions: *A*’s optimal plan depends on her inference about *B*’s optimal plan, which in turn depends on *B*’s inference about *A*’s optimal plan. The paradox arises because, in social *interaction*, plans are entangled (Table 2).[Table tbl2]

### We-Reasoning: Social Interactions and Joint Actions

There is a natural and intuitive way to escape the problem of mutual prediction. According to this approach, we should not imagine each person asking: *what do you think that I think that you think …* without limit. Instead, each participant in an interaction should ask: what do *we*, considering ourselves composing a single supra-agent (a team, a group), think we should do? Crucially, this perspective involves assigning beliefs, desires, and reasoning processes, not merely to individual members of a group or team; but to the group or team considered as a single agent. Indeed, extending talk of mental states not just to individuals but to groups is familiar in everyday discourse. We speak of a jury believing a criminal to be guilty; a parliament deciding to pass a law; or Real Madrid aiming to play entertaining rather than defensive soccer. In such contexts, we are saying more than that each individual in the relevant group has a particular view (and indeed, individual jurors, for instance, might privately dissent). We instead view the group as a single collective agent (Gilbert, 1987).^12^

Using this viewpoint, then, one way to address the problem of mutual prediction is (a) for the two or more interacting individuals to consider themselves as a single collective agent of which each is a member; (b) for each individual to ask how this collective agent should reason and what it might decide; and (c) for each agent to choose their individual courses of action according to the imagined recommendations of the collective agent.

This type of approach is often referred to as *we-reasoning*, and has been widely discussed and developed in related but distinct ways, across philosophy, economics, and psychology (e.g., Bacharach et al., 2006; Gallotti & Frith, 2013; Gilbert, 2006; Hakli et al., 2010; Sugden, 2003; Tenenbaum et al., 2011). Indeed, as noted above, we-reasoning, under the label of “shared intentionality,” has been proposed as a distinctive feature of human cognition (e.g., Tomasello & Rakoczy, 2003). Of particular interest, in terms of underlying cognitive mechanisms, is the concept of shared or joint attention, in which it is common ground that people are attending to the same object or event. An extensive program of research in social psychology has found that people preferentially devote cognitive and emotional resources to items they believe to be “co-attended” with others (e.g., Boothby et al., 2014; Shteynberg & Apfelbaum, 2013; Shteynberg et al., 2014). Shteynberg et al. (2020) develop this viewpoint further, with notions of collective attention and collective learning, which seem to be crucial incoordinating group behavior. These ideas help clarify the underpinnings of we-reasoning: for a group of people to share thoughts and intentions, they need minimally to be collectively attending to the same information and collectively attempting to solve the same problem.

The notion of we-reasoning does, though, raise a substantial theoretical challenge: How can an apparently mysterious collective agent arise from the thoughts and actions of individual agents?

One clue concerning how to proceed is to consider the status of the conclusion that is sought: for example, that given relevant background knowledge, situational factors, and the nature of the action, *we* should agree that this is an act of, say, giving (*G*) rather than showing (*S*). But presumably *we* can only derive conclusions based on premises that *we* endorse: that is, from information that is in common ground (Clark, 1996).^13^

So, for example, suppose that it is in common ground that *A* just said to *B*: “I’ll be really interested in your thoughts about my new book,” and handed *B* the book after signing it with a fountain pen. In the light of this information, it seems overwhelmingly likely that *A* is giving the book to *B*. Indeed, this would surely be the presumption of a disinterested third party observing the interaction. If this inference is itself in common ground, then the interpretation *G* will also be in common ground. Thus, *B* can safely thank *A*, and hold on to the book, without fear of social disaster.

The reason-based explanation that spells this out more fully turns out to be surprisingly subtle. To interpret the intention behind *A*’s action, *B* must ask: in the light of the knowledge of this situation that is in common ground for both of us, what is the natural interpretation of this action? That is, what would a disinterested third party, sharing this common ground, conclude about *A*’s intention (*G* or *S*?). Similarly, in generating the action, *A* should follow the same reasoning to assign an intention to her own action. Thus, in choosing her action with the aim that the social interaction will run smoothly, *A* must ask: what action can I take such that we would both agree that the intention is *G* (or *S*, depending on whether *A* wants to give, or merely show, the book to *B*).

One way to put this point is to see social interactions as a type of shared mental activity (even if the action, such as giving or showing, is conducted by only one partner).^14^ There may, of course, be additional layers of social complexity, which will be outside the scope of we-reasoning. For example, in handing over the book for inspection, *A* may hope to impress *B*, but probably does not want *B* to realize this. Similarly, *B* may infer that *A* is self-important, but assume that this is definitely not in common ground. Or *A* may suddenly fear that *B* will see her actions as boastful. The shared mental activity, though, is what makes this a social *interaction*, rather than simply one person attempting to manipulate or understand another.

The behavior arising from such shared mental activity can then be interpreted via the inverse planning that we described above (Baker et al., 2009), but now at the level of the collective agent. Thus, the intention of the action (say, *G*) is derived by inferring what we (i.e., the collective agent with the common ground of *A* and *B*) would have to jointly intend, were we to decide to choose that *A* perform this action. Given that *A* has performed this action, we can then infer from common ground that we must have had that intention. In particular, if *B* can infer that this intention is in common ground (in the light of the observed action—e.g., handing over the book in a particular manner), then *B* can infer that *A* has that intention *G*. Anything that is in common ground must be known to both *A* and *B*, and hence can be inferred by *B*.

From this “joint planning based on common ground” standpoint,^15^ it is possible to explain *why* certain features of the action would suggest one interpretation or the other. For example, opening the book and writing something in it makes sense according to the plan, “*A* signs and gives a book to *B*.” But it is utterly unnecessary in the light of the plan, “*A* shows *B* a book for a moment.” By contrast, pointing out a curious detail of the cover while handing over the book is a natural part of a plan in which *A* allows *B* to inspect a small and difficult-to-see quirk of the cover design. But it is unnecessary in the action of making a gift. Although explicating this reasoning is conceptually subtle, the degree to which it is reliable can in practice be assessed straightforwardly. Any disinterested third party with an understanding of the relevant common ground can decide how plausibly the conclusion follows.

The focus on the importance of common ground provides a basis for a reason-based understanding of some of the subtleties of everyday social interactions. Suppose, for example, that *C* whispers to *B* that *A* is giving the book away to everyone and that *B* should be ready to receive it. In this case, *B* now knows *A*’s intention to give the book to *B*, and *A* of course also knows her own intention to do so. But the intention is not in common ground: for example, *B* does not know that *A* knows that *B* knows it. And this is crucial. Suppose that *A* were to casually pass a copy of the book to *B*. *B* cannot then say, “Oh, such a generous present!”—indeed, this will be viewed by *A* as presumptuous, and by *B* as a terrible faux pas. Rather, *B* must wait until *A* signals that the gesture is a gift that *will* be in common ground, so that the information to underpin the conclusion that “this is a gift” can be inferred by we-reasoning. Furthermore, suppose that *A* overheard *C*’s whisper. Nonetheless, *A* will feel consternation at *B*’s claiming the book “all too readily”—because although *A* knows that *B* is expecting the gift, *B* behaves as if this expectation is in common ground (i.e., derived from we-reasoning), where it is not.

The invocation of common ground, rather than layers of mind-reading the other who is mind-reading us, breaks the circularity of the paradox of social interaction. But it raises puzzles of its own. In particular, from an individualistic perspective, the challenges of deciding definitively whether some piece of the knowledge, *K*, is in common ground might seem to involve a particularly complex mind-reading challenge: *A* might seem to need to establish that both she and *B* know *K*, that they both know that each other know *K*, and so on. If common ground is viewed as depending on an infinite hierarchy of beliefs about beliefs about beliefs, then establishing common ground itself sinks into paradox.

How can we possibly establish an infinite sequence of nested epistemic claims? In line with other authors (e.g., Clark, 1996; Shteynberg et al., 2020), we stress that common ground cannot be rooted in inferences from individual knowledge. For example, Clark (1996) notes that we may sometimes directly infer information common between us (e.g., an announcement over a public address system; a salient object that is in plain view for us both; “well-known” facts, e.g., that we learn at school; norms routinely used by everyone in our community; or facts that anyone in our company must have learned in their induction, etc.). Thus, common ground can often be inferred directly, and the infinite sequence of nested epistemic claims can then be inferred as a consequence. Thus, *A* may assume that she and *B* have common ground that Paris is the capital of France, from the fact that capital cities are routinely learned at school; and then can infer that *B* knows this, that *B* knows that *A* knows this; that *B* knows that *A* knows that *B* knows it, and so on.^16^

### Virtual Bargaining

We noted earlier that the social sciences have a long tradition of explaining the behavior of groups in terms of interactions between the individuals of which they are composed. Indeed, this viewpoint is particularly strong in psychology, where social behavior is explained through the lens of individual minds. From this point of view, a crucial theoretical challenge is to establish the preferences or goals of any postulated supra-personal entity (the “we” in we-reasoning), perhaps from the preferences and goals of the individuals of which it is formed.^17^

One approach is to assume that the “supra-agent” is benevolent toward its members, perhaps aiming to maximize their summed utilities (e.g., Bacharach et al., 2006; Colman & Gold, 2018). This approach faces the problem of interpersonal utility comparisons (Diamond, 1967; Elster & Roemer, 1993; Weymark, 2016) as well as the difficulty of taking into account the possibility that individuals never completely abandon their own objectives for those of the group.

A more fundamental problem arises from social interactions in which power is very unbalanced. Suppose, for example, *X* is a gangster and *Y* a downtrodden and fearful side-kick. If *X* passes a valuable stolen watch to *Y*, *Y* will interpret this as “showing” and return it right away. But if *Y* hands a stolen watch to *X*, *X* will keep it. Of course, if *X* is very rich and *Y* is very poor, summed utilities might be maximized if the watch is owned by *Y* not *X*, and a “benevolent” team might thus choose to assign the item to *Y* (Bacharach et al., 2006). But this will be irrelevant for understanding the social interaction between the gangster and side-kick: this particular “team” will agree that the watch will go to the less-needy but all-powerful gangster. Power relations, although of huge importance in understanding social interactions (Guinote & Vescio, 2010), are orthogonal to questions of unconditional aggregate utility maximization.

**Figure 1 fig1:**
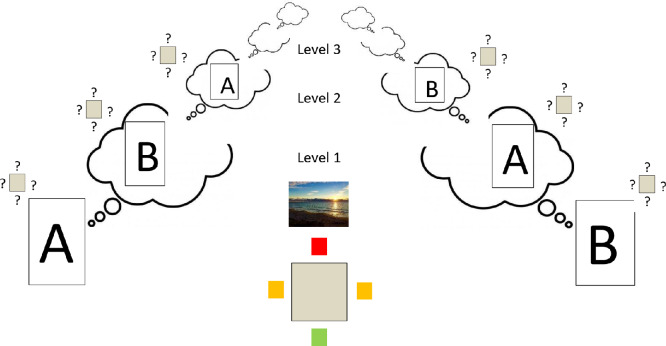
The Paradox of Social Interaction *Note*. Two people, *A* and *B*, attempt to coordinate their thoughts and behavior by reading the mind of the other. Here, *A* and *B* must sit opposite each other when at a table with a sea view on one side. Who should take the chair with the sea view (green square), forcing the other to have no sea view (red square)? In many circumstances, probably neither should, allowing both to have a partial sea view (yellow squares). Sometimes, one person (rarely seeing the sea) might have a much stronger preference, which perhaps both *A* and *B* might respect. The social interaction will go smoothly if *A* and *B* make “complementary” choices, rather than both attempting to sit in the same chair (especially the chair with the sea view). The problem for mind-reading accounts is that each person is viewed as attempting to read the mind of the other, who is trying to read their mind, and so on, indefinitely. Focusing on the left-hand side of the “triangle,” consider *A*’s reasoning. *A* will attempt to second-guess *B*’s choice (Level 1); but *A* knows that *B*’s choice will depend on *B* second-guessing *A*’s choice; and so on. *B*’s reasoning, on the right-hand side of the triangle, is analogous. In each case, the question of what to do requires second-guessing what the other will do, continually pushing the “question” to a higher level, without ever reaching a resolution. See the online article for the color version of this figure.

**Figure 2 fig2:**
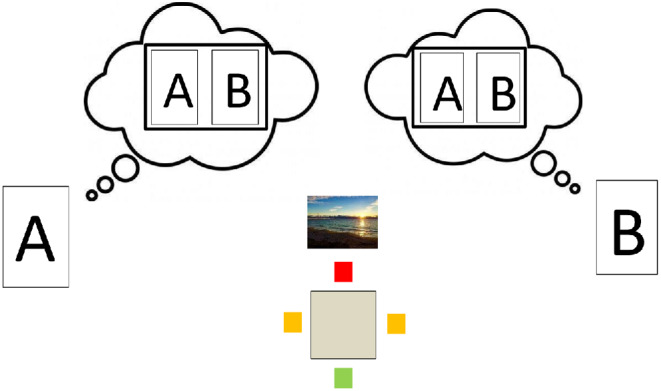
We-Reasoning *Note*. Both *A* and *B* reason about how the two of them would behave as a group: that is, they both ask “how would we interpret this action? In one version of we-reasoning, team reasoning (e.g., Bacharach et al., 2006), both people ask themselves what choice they would make if they were a “team” with the same beliefs and goals. The virtual bargaining approach (e.g., Misyak et al., 2014) asks, “what would we do, if we could discuss and negotiate?.” These approaches involve each of *A* and *B* attempting to reason about the same thing, for example, the result of an imagined interaction between *A* and *B*. Thus, the problematic regress is avoided. See the online article for the color version of this figure.

A further complication arises when, as is often the case, *A* and *B* must both coordinate their behavior but have partially conflicting interests. Suppose *A* and *B* are at a beach-side restaurant with magnificent sea views (Figure 1). A square table at which they must be seated has one chair facing the sea with an excellent view (Good), but the opposite chair has no view at all (Bad). The other two chairs face side-on, both having a partial sea view (Medium 1 and Medium 2). Let us assume, plausibly, that *A* and *B* must sit opposite to each other (the table is quite small). So, either one person has Good and the other Bad or they both get medium outcomes (Figure 2).[Fig fig1] [Fig fig2]

Note, first, that models based on the Theory-Theory or Simulation-Theory of Mind, and prediction-based models, will be at a loss to deal with this scenario: the paradox is in full force. Either theory of mind-reading will propose that *A* tries to model *B*’s reasoning (presumably to take the opposite chair, to avoid social disharmony); but then *B* will be attempting to model *A*’s reasoning—and we have gone in a circle. And according to prediction-based models, both people are engaged in a deadlock of mutual prediction.

How far does we-reasoning take us? Let us start by considering what can be inferred from common ground. There are three possible joint outcomes, with payoffs [*U*_*A*_ = *H*; *U*_*B*_ = *L*], [*U*_*A*_ = *L*; *U*_*B*_ = *H*] and [*U*_*A*_ = *M*; *U*_*B*_ = *M*]. So, *A* and *B* are facing a coordination problem: ideally, they would identify the same option and smoothly instantiate it. But can they avoid choosing incompatible options leading to social confusion and embarrassment?

We suggest that the following line of argument presents a promising theoretical direction. Notice that deciding which of the outcomes is most appropriate requires a mechanism for trading off *A*’s and *B*’s conflicting interests. If *A* and *B* were to communicate, then they might be able to decide on a trade-off by negotiation.^18^ But in many cases it may be sufficiently obvious to *A* and *B* what conclusion this negotiation would reach that communication is unnecessary. Thus, we have a candidate mechanism to guide social interaction in the absence of communication—both parties need merely implement what they would have negotiated. We have elsewhere called this mechanism *virtual bargaining* (e.g., Melkonyan et al., 2018, 2021; Misyak & Chater, 2014; Misyak et al., 2014).

What, then, is the basis for the judgments concerning the “obvious” agreement? Note that, if such an agreement is to follow from reason-based explanation, any conclusion concerning the outcome of virtual bargaining must be in common ground: both players must be able to infer that they both know it, know that the other knows it, and so on. Imagine, in our sea view example, that *A* and *B* are rival business people, with no fellow-feeling. Nonetheless, person *A* has the “trump card” of living in the middle of a continent, and not having seen the sea for many years; whereas person *B* is a local who sees the sea every day. Both would prefer the sea view; and each has researched the other’s background—so both know about *A*’s trump card. Nonetheless, if this is not in common ground (i.e., they do not know what background research the other has done), then it would be socially highly inappropriate for *A* to make a dash for the chair with the sea view, and *B* would react with consternation.

Reflection on examples of this kind suggest that virtual bargaining must derive the conclusion that “if we could communicate, we would agree to *this*” from common ground. The decisive inferential step is moving from this common-ground hypothetical to each carrying out the inferred action.^19^

As in the examples above, what counts as the “natural” bargain in the light of common ground can, in general, be assessed by third parties, given that common-ground information. Indeed, when *A* and *B* are assessing what bargain they might hypothetically reach, they are attempting to adopt such a dispassionate stance. If they are able to do this successfully and a unique natural bargain emerges, then a harmonious interaction is likely to result. In practice, a full range of cognitive shortcomings (e.g., self-serving biases; projection of one’s own interests to the other; the curse of knowledge) have the potential to derail this process and may lead to a dissonant social interaction. But, we suggest, social interactions can be understood as striving toward the objective of implementing a virtual negotiation based on common ground. Indeed, this framework is especially helpful in understanding attempts to forestall or repair dissonant interactions, for example, by *A* saying “wow, I’ve not seen the sea for 20 years” as *A* and *B* walk towards their table or stand at the table struggling with the choice of seats.

Note, too, that the virtual bargaining viewpoint naturally captures the impact of power asymmetries. If gangster *X* and side-kick *Y* approach the restaurant table, *X* will head directly for the best seat, and *Y* for the worst. Both can infer that this would be the outcome were they to bargain (because *X* has overwhelming bargaining power); hence, they simply implement the result of this hypothetical bargain without needing to communicate.

How far can virtual bargaining be modeled formally? In specific contexts, where common ground, including payoffs, is well defined (e.g., in laboratory experimental games or economic transactions), this is possible. Indeed, we have developed a game-theoretic model, with equilibrium conditions (“feasible agreements”) that possible bargains must fulfil (e.g., Melkonyan et al., 2018, 2021; Misyak & Chater, 2014; Misyak et al., 2014). To choose between feasible agreements and alight where possible on a single virtual bargain, the model needs to draw on a theory of bargaining (after all, this is what virtual bargainers are attempting mentally to simulate). In economics, there are various theories of bargaining where the “best” bargain depends only on well-defined “utilities” of the players. Melkonyan et al. (2018, 2021) use the most well-known theory, Nash bargaining^20^ (Nash, 1953), which selects the option that maximizes the product of utility gains for the players for the bargain (over and above the utilities that would be obtained if bargaining failed). But other theories of bargaining could be used instead.^21^

But for other aspects of informal reasoning and argumentation which operate in an open-ended and only partially understood world, complete and precise formal analysis of virtual bargaining will often not be possible. Indeed, just as in common-sense reasoning more generally (e.g., Fodor, 1983; Harman, 1986; Oaksford & Chater, 2007), the range of factors that can shape the natural bargain is limitless. Nonetheless, our intuitions about which bargain would be reached in a hypothetical negotiation are often quite clear cut. Consider the following variants of the chair-choosing scenario:

i Person *A* is the CEO and *B* a new employee. The natural agreement will probably be that *A* takes the better seat and *B* the worse, and this may be implemented without comment. Interestingly, the CEO can “reopen” the negotiation to her potential disadvantage, for example, by edging toward, or just selecting, one of the “Medium” chairs. For the new employee to do so would be a social gaffe.
ii *A* and *B* are good friends; it is in common ground that neither will be happy if the other is disadvantaged. They may naturally both select “partial view” seats.
iii *A* and *B* hardly know each other but are keen to make a good impression (i.e., to preserve social harmony). Thus, both know that it will be a faux pas to try to “capture” the best seat (i.e., they do not have sufficient common ground to reach any particular asymmetrical outcome) and hence they both align on the “partial view” seating arrangement. Here, unlike in (ii), there is no concern about the other’s well-being. Now it is possible that either *A* or *B* may, like the CEO above (i), attempt to renegotiate to their own disadvantage, for example, by gesturing the other towards the seat with the sea view. If accepted, this may be interpreted as a friendly act. On the other hand, it may be viewed as overly ingratiating and insincere, perhaps as attempting to imply concern for the other’s well-being, which seems to have no basis.

The aim of our arguments is to illustrate the *structure* of the social reasoning in which we routinely engage—the types of considerations that matter, the importance of what is in common ground, and the we-reasoning and virtual bargaining through which people strive to create implicit agreements underscoring harmonious social interactions. Once this structure is clear, it is possible to define precise formal models and make experimental predictions for specific situations, based on assumptions about the social participants’ common ground (including their action alternatives and their payoffs; e.g., Melkonyan et al., 2018, 2021).

### Implications

We have argued that social interaction typically involves a mutual adjustment of thought and behavior and that a reason-based explanation of this process cannot operate by a process of mutual prediction, on pain of circularity. Instead, coordination is achieved, where it can be achieved at all, by a process of simulated bargaining between the people involved in an interaction, where the outcome of that simulation is based only on their common ground (and is not based on any knowledge that is provided only to one person). Here, we consider implications and applications of the paradox and its solution using virtual bargaining.

#### The Paradox of Social Interaction and Theories of Shared Intentionality

Psychological theorizing has drawn extensively on philosophical analyses of shared intentionality and related concepts. How does the present discussion, both of the paradox of social interaction and of the proposed solution via virtual bargaining, relate to such analyses? Most fundamentally, the focus here is on how people are able to alight upon, and carry out, a coordinated plan—through simulating the process of agreeing on a plan based on the participants’ common ground. The variety of philosophical analyses typically have a complementary goal: to analyze what it *means* to have a shared plan, rather than resolving the problem of how such plans are selected. Indeed, the problem is often sidestepped by assuming that a common plan is articulated linguistically, for example, “let’s move the table” or “we’re going to the store.” Indeed, Tuomela (2005) explains his account metaphorically by imagining a publicly visible plan (e.g., of roles and responsibilities in cleaning a park), to which participants can equally publicly “sign up.”

It is clear, though, that much coordinated action occurs with little or no explicit verbal interaction. Even where verbal agreements are formulated, these are typically incomplete. Thus, when two people walk to the coffee shop together, there is no explicit agreement to go at the same pace, and not to hop, walk backward, stop unexpectedly, make a long phone call, and so on. But these “ground-rules” and many more are nonetheless implicitly agreed, as is evident by the fact that to violate one of them would require an explanation or apology (e.g., Garfinkel, 1967).^22^

#### Pluralistic Ignorance

Interesting implications for the phenomenon of pluralistic ignorance emerge from the fact that the virtual bargain depends only on common ground (e.g., Prentice & Miller, 1993). Thus, everybody at a party may believe that it is in common ground that students at a particular university like to binge drink, and disapprove of anyone who does not, even though each student may actually disapprove of binge drinking and privately envy those who avoid it. This has the perverse consequence that each individual may subscribe to the presumed result of the virtual bargain and affirm that “we plan to drink heavily tonight” even though each person would rather not. Indeed, it might be the case that were the students to have a discussion, they might discover that they would all prefer to play table tennis or watch a movie. In these circumstances, virtual bargaining would have failed correctly to simulate the outcome of real bargaining—because people are unaware of each other’s true preferences. Yet there is also a second possibility: that virtual bargaining might *correctly* predict that actual discussion would favor heavy drinking over a movie, even though this might violate each person’s individual preferences, because contributions to the discussion would be shaped to conform with the expected consensus. That is, in some circumstances, real bargaining would lead to the perverse outcome because people would be unwilling to reveal their private preferences through pressure to conform; and if people correctly mentally simulate this, then their virtual bargaining will have the same perverse result. Thus, virtual bargaining can become a self-enforcing mechanism in contexts of pluralistic ignorance: simulating the presumed agreement suppresses “honest” expression of preferences and (a) makes that agreement likely if overt discussion were to occur and (b) makes overt discussion less likely, as the result is presumed to be already known. Indeed, we suggest that virtual bargaining may be crucial to the stability of a variety of group behaviors that are out of line with individual preferences. Few of us enjoy being on the losing side of a debate; so mentally simulating the outcome of such a debate may cause us to stifle our dissent, so that the debate never takes place at all.

#### The Nature and Origin of Common Ground

As we have noted, virtual bargaining and we-reasoning more broadly between two or more people can only proceed on the basis of their common ground. This raises the questions of what common ground can be assumed, how information enters common ground, and how people are able to establish what is in common ground when uncertainty prevails.

The now-familiar problem of infinite regress rules out the possibility of inferring common ground from private knowledge. Thus, to adapt an example from Clark (1996), if *A* privately knows that *B* is from New Zealand, then *A* will likely assume that *B* knows the name of the current Prime Minister of New Zealand (among many other things). But this conclusion is not in common ground. Indeed, *B* has no way of knowing that *A* knows it, because *A*’s premises are private. But if *B* simply tells *A* that she is from New Zealand, then this information is put in common ground, and the conclusions may be in common ground also.

The requirement that conclusions in common ground can only be based on premises in common ground has surprisingly strong implications. A notable feature of everyday world knowledge is that it is a densely interconnected and interdependent network (e.g., Fodor, 1983; Quine & Ullian, 1978), such that typical inferences (e.g., from being a New Zealander to almost certainly knowing the name of the current Prime Minister of New Zealand) depend on background knowledge about what New Zealand is and what a Prime Minister is, which themselves depend on further knowledge about geography, politics, and much more. Indeed, each piece of knowledge depends on further background knowledge, and so on, without end.^23^

Thus, to avoid infinite regress, entire *systems* of background beliefs must therefore be treated as common ground by all parties. So, rather than attempting to build up common ground through individual knowledge, it seems unavoidable that general background knowledge, at least, is taken for granted as common ground. From this point of view, common ground may, perhaps counterintuitively, be more psychologically basic than individual knowledge. That is, it may require active cognitive processing for a person to recognize that information that they individually know is not in common ground, and to take account of this fact. Such processing is often effortful and not fully carried out. Indeed, this provides a new perspective on the so-called “curse of knowledge” (Camerer et al., 1989) in which people consistently assume that others know what they themselves know. The curse of knowledge can lead to poor outcomes in economic bargaining (Camerer et al., 1989); to difficulties in reasoning about false beliefs for both children and adults (Birch & Bloom, 2007); and to a tendency for the sender of a message to underestimate how difficult the receiver may find it to decode (Newton, 1990). Specifically, we suggest that the curse of knowledge should be viewed as following from the unavoidable default assumption that one’s knowledge is in common ground.

The ability to communicate and interact successfully with others will depend, of course, on how far these assumptions of common ground are correct. If people’s assumptions about common ground are largely aligned in a specific context, then they will interpret each other’s behavior in the same way, and social interaction is likely to proceed smoothly. The importance of shared common ground in coordinating social behavior may help explain the strong positive affect associated with perceiving that we have a shared perspective with others, and the strong negative affect when we do not. Crucially, as Shteynberg et al. (2020) point out, this positive affect is generated not merely by the shared common ground, but by shared attentional focus being directed on the existence and relevance of this common ground. Similarly, the subjective experience of I-sharing—the momentary subjective experience of sharing a sense of self with another person—may reflect awareness of common ground assumptions being strengthened (e.g., if we face a common challenge or trauma, mimic each other’s behavior, sing or dance in synchrony, and so on, Pinel et al., 2006). The subjective perception of such cognitive alignment with others is one way to understand the important concept of “shared reality” (Echterhoff et al., 2009; Higgins, 2019; Rossignac-Milon et al., 2021).

Perhaps even more basically, the drive to establish common ground—and hence facilitate future virtual bargaining that can coordinate social behavior—may provide a motivation for the enormous amount of time people spend in apparent “idle” conversation. Thus, although some anthropologists have assumed gossip to be an analog of grooming in nonhuman apes (e.g., Dunbar, 1998), it may be more appropriate to see conversation as establishing and solidifying the common ground for coordinating future behavior. This point is applicable particularly to what Clark (1996) calls *personal* common ground, which refers not to general background knowledge shared by the community at large, but to information shared by specific pairs or small groups of individuals. Building common ground through conversation and shared experiences seems to be a crucial part of building relationships, whether in friendships, romantic relationships, business partnerships, sports teams, or groups of workers.

#### Affiliation and Groups

If social interactions are coordinated by tacit agreements, then the degree to which we will wish to enter such interactions will depend on our expectations about how easily tacit agreements can be formed, and how far they will be followed rather than flouted. The ability to form such agreements, as we have seen, will depend crucially on common ground—so that interactions will, other things being equal, proceed more smoothly for people with similar backgrounds, training, experiences, and so on. Thus, in a telecoms company, managers with a background in sales may find it easier to form tacit agreements with each other than with the engineers, and vice versa. This may lead to increased levels of liking, friendship, and further interactions within rather than between groups, potentially reinforcing the differences in common ground between sales people and the engineers. On the other hand, lack of diversity may reduce the range of knowledge and experience available and hence reduce the quality of the resulting decisions.

A further interesting topic for future research is whether such effects are amplified by attribution errors arising from the curse of knowledge. If people in different groups tend to overestimate their common ground, then failed interactions are more likely to be attributed to “bad faith” on the part of the members of the other group. For example, if an engineer gives a technical explanation that is incomprehensible to a manager, this might arise because the engineer wrongly assumes this relevant technical knowledge as common ground. But it might be interpreted as a deliberate attempt to show off, obfuscate, or otherwise be unhelpful. This may be one of many forces maintaining in-groups and driving them away from out-groups, and more broadly causing people to affiliate with people they perceive to be like themselves. It might also suggest that contact between members of different groups may reduce such effects, especially if such contact leads to successful social coordination and collaboration (Paolini et al., 2018). Indeed, recent experimental work suggests that even shared attention (e.g., to a brief video arguing for or against evolution by natural selection) may selectively increase liking for another person (Haj-Mohamadi et al., 2018). Perhaps tellingly, this effect only occurs if the shared information is congruent with a person’s beliefs (i.e., whether the person believes in natural selection or creationism). Where the new information is not congruent with their prior assumptions, the person is blocked from encoding it as common ground between them and their partner because they do not believe it themselves.

#### Relation to Social Norms

As we have seen, flexibility is a hallmark of human social interaction. From this standpoint, it seems misleading to think of the social life as guided predominantly by “norms,” viewed as fixed rules of behavior like the rules of chess.^24^ Very subtle changes in the environment, or how a person acts or reacts, can completely change a social situation, depending on the underlying implicit agreements in play. Suppose two people approach a supermarket check-out at around the same time. If one person slows and waves to the other to go first, this may be interpreted as politely and helpfully breaking the deadlock. But suppose that a faster moving young person dashes slightly ahead of a slower moving older person. Now, if the older person slows and waves the other through, this may signal exasperation, or even hostility. The act of signaling draws attention to the deadlock and that the younger person has “resolved” it, without agreement, to their own advantage. Slight exaggerations of the stopping and waving may intensify the communication of frustration. The younger person then faces the difficult question of whether to acknowledge the gesture, as if it were meant sincerely; to ignore it; or perhaps to apologize and let the older person go first. Such interactions are better viewed as complex, ad hoc, improvizations, rather than the playing out of a game according to fixed rules (Bertinetto & Bertram, 2020).

Still, though, many interactions are repeated, so that particular patterns of behavior will become increasingly conventionalized (although any conventions will always be open to challenge, irony, exaggeration, subversion, and so on). Indeed, it is interesting to speculate that social norms may best be viewed as emergent patterns arising over time from specific improvized interactions, by analogy with the way in which many linguists and psychologists in the “usage-based” tradition see grammatical patterns as arising through gradual entrenchment of, and generalization from, specific uses (Hopper & Traugott, 1993; Tomasello, 2003). This viewpoint has the implication that virtual bargaining may be essential to the development of human communicative and social norms, and thus that species without the ability to engage in virtual bargaining would be unable to develop a complex culture.

#### The Importance of Scripts, Roles, and Responsibilities

Many social situations (greetings, being seated or served by a waiter, going through a religious ritual, receiving a certificate at a school assembly) are governed by something close to standardized “scripts” (Schank & Abelson, 1977), in which different participants have distinct roles and responsibilities. For example, a waiter is expected to ask a customer for their order, not the reverse; the waiter is responsible for passing the order to the chef, and so on. Where scripts are in common ground, they can, of course, substantially simplify the problem of social coordination—but a coordinated social interaction requires continual negotiation (and, often, repair) for the script to be “performed” successfully. The complexity of such coordination requires that most such negotiation cannot be mediated by language, but must be predominantly “virtual;” and the need for different parties to arrive at the same solution, out of multiple possibilities, raises, as ever, the paradox of social interaction: that each party is attempting to predict, and coordinate with, the actions of the other. So, for example, both the waiter and customer need to agree which table the waiter is attending to (the waiter may pass near a table, for example, merely to allow other customers to pass). They need to agree, too, when it is appropriate to request, or to place, and order (e.g., not if the waiter approaches the table with a dustpan and brush, after glass has been broken). Indeed, the virtual bargaining viewpoint meshes with a dramaturgical perspective on social behavior, as the joint creation of a shared “performance” (Goffman, 1959).

#### Deciding Who “We” Are

We-reasoning, including virtual bargaining, begins from presumed common ground about who “we” are—that is, who the parties are in the social interaction. Simple physical presence is not, of course, sufficient. Thus, if one diner suggests going to a movie to another, a waiter who is clearing the plates, or a diner at the next table, would not be expected to be included in the invitation. Thus, an important part of any social interaction is ensuring that there is common ground among participants that they *are* participants.^25^

Misconstruing who is, or is not, engaged in a particular social interaction can itself be a source of social confusion and embarrassment—as when we wave back at someone who was signaling to someone else; or when taking a seat that is actually being offered to someone else. An open theoretical question concerns how one’s legitimate participation in a social interaction is itself commonly agreed. Assuming that this is itself achieved via virtual bargaining seems to raise the specter of circularity, because such bargaining itself presupposes an agreement about who is involved in the agreement. We suspect the circularity may be more apparent than real: that the question of who is *in* the virtual bargain is really part of the bargain itself (just as the terms of a business contract include the identities of the participating firms and individuals).

Nonetheless, the issues here are subtle. For example, consider informal and apparently tacit conventions, such as those described by Sugden (1989): in a Yorkshire fishing village, anyone could mark a pile of driftwood as theirs (for later collection) by placing two stones on it. A villager could not, presumably, violate the rule and take driftwood for themselves merely by declaring that they were not part of the “bargain.” The normative force of the rule is that people in the relevant social group cannot simply declare themselves outside the bargain and hence not bound by it (even if they say that they do not object to others taking their driftwood). Relatedly, in some contexts, the question of who is party to a social interaction, and hence drawn into a virtual bargain, may be contentious: a fundraiser with a tin may wish to engage a hapless shopper, creating mutual expectation of a donation to a good cause; the shopper may avoid eye contact and pretend not to notice the fundraiser’s existence, to avoid “implicitly agreeing” to be part of any such bargain.

The question of who “we” are is likely to be closely linked to questions of social identity and group formation. A common identification that we are members of the same fishing community, regiment, religious group, political party, or nation may establish common ground that, in some ways at least, we are part of a common social unit, governed by an agreed social contract (e.g., to follow group norms, help each other, and so on). Thus, from this viewpoint, social identity (Tajfel, 1974) as being part of a group depends not just on self-identification as a group member, but on group membership being in common ground within the group (“we all know who we are”). This common ground is required for group members to coordinate effectively through virtual bargaining. Indeed, work in the minimal group paradigm has long shown that merely establishing in people’s common ground that they have been divided into two arbitrary groups (e.g., randomly given red versus blue t-shirts) leads to greater cooperation within rather than between groups (e.g., Brewer, 1979). This is often interpreted as indicating greater liking for, and hence pro-social behavior towards, in-group than out-group members. According to the present perspective, this same manipulation might also be expected to differentiate performance even in so-called Schelling games (Schelling, 1960), where people are each rewarded if they independently make the same choice as each other from a number of options (e.g., colors, dates, numbers, locations). As we have noted, in coordination problems of this kind, enhanced pro-social motivation does not improve performance. What is crucial is the ability to imagine what “we” would agree were we able to discuss what would be most natural (e.g., “choose the most common color,” “choose January 1st,” etc.). By priming or suppressing the “we” perspective through having people interact who are either in the same or a different arbitrary group, we would expect such inferences, and hence resulting coordination, to be enhanced or impeded. As far as we know, this prediction remains to be tested experimentally.

#### A New Perspective on Reciprocation

Many mutually beneficial social and economic interactions unfold over time, in which *A* helps *B* on some occasions, and *B* helps *A* on others. Indeed, some patterns of mutual help and the common expectation of such help are often a foundation for mutually beneficial relationships, rather than momentary transactions, from friendships, to life-partnerships, to business partnerships, and alliances of all kinds. Patterns of reciprocation are puzzling for traditional reason-based models of behavior. To see why, consider the well-known “Centipede” game.^26^ The game has a fixed maximum number of alternating turns (e.g., 10) between two players. On each turn, the player whose turn it is to move can either (a) give up $1 of her own money, so that the other player receives $3 or (b) stop the game. Over time, both players benefit from reciprocal interaction: each gives away a number of $1 sums and receives a roughly equal number of $3 sums. But if the game has a commonly known endpoint (e.g., each player can play just 10 trials), then a standard rational account is that no reciprocation is possible. Both players anticipate that the player with the final “turn” (say, *A*) will not send back a dollar (having nothing more to gain, because the other cannot reciprocate). They can then infer that *B* will not send back the dollar on the penultimate turn (knowing that *A* will not reciprocate on the final turn). Following this logic to the start of the game through “backward induction” leads to the conclusion that reciprocation can never start. Or, to consider a related example, consider a finitely repeated Prisoner’s Dilemma (PD; Embrey et al., 2018), in which each player can cooperate or defect on each turn (a simple version of PD is that, on each turn, both players simultaneously choose whether to give up $1 of their own money, so that the other player receives $3). On a single round of PD, of course, it is in each player’s interest to withhold their dollar (whatever the other does, this will leave them one dollar better off). But if PD is repeated, say, 20 times, then there is a substantial opportunity for mutual benefit by reciprocally cooperating. As in the Centipede game, rational agents seem unable to obtain such benefits, because of the logic of “backward induction”: both know that defection will occur on the last trial; so it is rational to defect on the second to last trial, and the third to last, all the way to the beginning of the game. So, the apparently substantial opportunity for reciprocation (I’ll help you, if you help me) appears inaccessible. In practice, people typically do engage in high levels of cooperation in both the Centipede and finitely repeated PD games, though sometimes ceasing cooperation near the very end of the game (Embrey et al., 2018; García-Pola et al., 2020).^27^

The virtual bargaining viewpoint outlined here suggests that reciprocation need not be treated as a distinct phenomenon (e.g., Fehr & Gächter, 2000b), but rather viewed as a special case of reaching a virtual agreement. Specifically, virtual bargaining can tell the players that, were they able to communicate, they would agree to be cooperative—that is, to keep paying across sums of $1 until the end of the game. While this hypothetical agreement is in common ground, each player may reasonably wonder: “what happens if I stick to the agreement, but the other “exploits” my doing so to their own best advantage?” In the Centipede and finitely repeated PD games, the best way to exploit someone who dutifully follows the virtual agreement is to continually cooperate until the very last turn. By exploiting the dutiful player only at the last moment, the selfish player gains all the accumulated benefits of mutual cooperation, and thus maximizes their own payoff. Crucially, however, the payoff to the dutiful player is still high, because they, too, gain the accumulated benefits of mutual cooperation, except when exploited on the very last turn. So the players can anticipate a good outcome for themselves from the agreement to cooperate to the end of the game, even where they suspect that the other might secretly intend to act selfishly. Each player can reason, “Of course, I can trust myself to stick to a virtual agreement to cooperate throughout the game; and even if the other player selfishly exploits me, my outcome is still good. So, this is a good agreement for me, whether the other person sticks to the agreement or behaves selfishly.” And this reasoning is itself in common ground, so it is commonly known to both parties that the agreement is mutually advantageous and resilient against exploitation (for a formal analysis, see Melkonyan et al., 2021).

This perspective is very different from many “algorithmic” perspectives on reciprocation, according to which people (or artificial agents) follow rules, such as tit-for-tat, which are assumed to have “evolved” either through cultural or biological evolution (e.g., Axelrod & Hamilton, 1981 and the large subsequent literature). By contrast, the present viewpoint sees reciprocation as a paradigm example of a “social contract” which will be in common ground to both parties, with common expectations that the contract will be fulfilled (and the expectation of complaint to, or even punishment of, the other, if it is not).

To see the difference, consider a (real) case in which *A* tidies *B*’s garden each week, and each week *B* gives *A* a bottle of wine. It turns out that *A* does not drink wine, and hence the “reciprocation” is entirely void, with the bottles simply accumulating undrunk. *A* nonetheless feels obligated to continue tending *B*’s garden, rather than violating the unspoken agreement (although clearly an agreement has arisen due to a misunderstanding). Suppose *A* were to discover that *B* does not drink wine (and *B* were to know that *A* had discovered this). Then, a conventional rational analysis would lead to the conclusion that *A* could abruptly stop giving wine and expect *B* to tend the garden as before (reasoning that *B* must enjoy the gardening for its own sake, as reciprocation is clearly not *B*’s motive). Yet this would almost certainly be viewed as extremely rude, and *B* might very well cease gardening at once. Instead, *A* would need to find some other (one hopes more successful) way of reciprocating—thus forming a new tacit “contract” with *B* (perhaps *B* could take produce or flowers from the garden). Or suppose *A* goes on a summer holiday for 2 weeks, and hence misses a “payment” of wine to *B*. In such novel circumstances, the two parties may not have sufficient basis for coordinating on the same virtual bargain, and hence disagreements may arise. Quite likely *B* will garden anyway, and anticipate two bottles of wine on *A*’s return. But *A* might provide just one bottle, assuming that a single bottle is a token of appreciation, rather than an implicit “payment” per week for *A*’s gardening. There would be at least the possibility that *B* might feel affronted if *A* did not provide some extra token of appreciation on her return, even if the token is entirely unwanted. Indeed, irritation and even anger are likely to be common responses when virtual bargaining fails.

These patterns of thought and behavior are intuitively natural and make sense if both parties think they have entered a tacit agreement. But they do not follow from any algorithmic account of reciprocation, for example, following a rule such as tit-for-tat.^28^ Furthermore, this viewpoint helps explain why people may *reject* help from another, when this is apparently against their interests—because accepting help may, in some contexts, signal tacit acceptance of a mutual bond of reciprocal support to which one party may be reluctant to sign up (as when one accepts an offer of help from the mafia, Gambetta, 1996).

#### Links With Other Types of Contract-Based Theorizing in Psychology

The present account of how “social contracts” are improvized in the moment has interesting parallels with other ideas in psychology. In the psychology of language, Brennan and Clark (1996) propose that “conceptual pacts” coordinate moment-by-moment communication (a conceptual pact might be: “let’s call this odd-shaped tangram pattern ‘the rocket’” or “in this conversation, ‘Ali’ refers to my friend rather than the legendary boxer”). More broadly, social scientists frequently talk of people negotiating meanings, identities, and relationships (e.g., Thomas et al., 2011)—but of course much of this negotiation is not explicit and may perhaps usefully be considered as stemming from virtual bargaining.

A second connection focuses on a putative psychological contract between employees and companies (Argyris, 1960; Rousseau, 1995; Rousseau & Shperling, 2003). Again, virtual bargaining provides a potential route to understanding how such psychological contracts can become established without, or with a minimum of, explicit communication. We believe that it is crucial to understand such contracts as jointly recognized and understood. Thus, for example, if a worker is not treated as they expect by their employer (e.g., their hours and pay are reduced without prior notice), their outrage is, we suspect, not merely that the employer has deviated from the employee’s understanding of their implicit agreement. It is rather that the employer has knowingly and willfully violated an implicit agreement to which they had both “signed up.” The employee thus feels a sense not merely of having been maltreated, but betrayed. It may therefore be useful to recast the important literature on psychological contracts from the viewpoint that psychological contracts are joint contracts (or at least are perceived as joint contracts), rather than being mentally represented by a single individual.

A third related theoretical idea is what Rousseau (1989) called “implied” contracts. These are implicit rules by which society at large guides and governs relationships (e.g., between employee and employer), whether or not these are mentally represented by either party. What is the status of such implied contracts? We suggest that they may be analogous to the rules governing language, which are not explicitly formulated by any individual speaker but are distributed through a linguistic community. The parallel with language also suggests that implied contracts, and implicit social norms more generally, may become established over time through the gradual accumulation of virtual bargains each of which deals with specific circumstances. Each new virtual bargain will build on those that have gone before, via processes of entrenchment and generalization, potentially producing an increasingly conventionalized, rule-like system.

#### Relation to Moral Reasoning

These considerations suggest, more broadly, that social interactions are governed by tacit agreements with a “moral” dimension. That is, a commitment to the “rightness” of the tacit agreement, having an obligation to uphold it, and accepting the appropriateness of chastising or punishing those who violate it without good cause, is *part* of committing to the agreement itself. Indeed, when people violate tacit agreements, even in low-stakes social interactions concerned with seating preferences, borrowing pens, or handing each other books, the reaction of others can range from mild irritation to outrage. More broadly, a model of social interaction founded on mutual tacit agreements, rather than second guessing the thoughts and actions of another (who is second-guessing one’s own), explains the inherently *normative* nature of social behavior, and that social norms are typically viewed as common ground between participants.

From a traditional individualistic perspective, it is natural to think of people as focused primarily on the consequences of their own and other people’s actions. From this point of view, we might expect people to praise and reward the actions of others that have positive consequences for them and to punish actions that have bad consequences for them (which might be the foundation of reciprocal cooperation, Fehr & Gächter, 2000a). But the examples above indicate that people are also greatly concerned with people following tacit (and indeed explicit) agreements. From this point of view, the human ability to engage in coordinated social behavior may be continuous with our sense of the “appropriate” way to behave (March & Olsen, 2008), a viewpoint that is consistent with developmental evidence (e.g., Tomasello, 2019). Indeed, if rules of local social interaction are continuous with wider moral thinking, then virtual bargaining may provide a starting point for understanding moral thinking more broadly, and especially those that naturally flow from contractarian approaches to ethics (e.g., Gauthier, 1986; Scanlon, 1998).

#### Virtual Bargaining and Verbal Communication

We have argued that virtual bargaining provides a mechanism through which people can coordinate their behavior without communication. But even where verbal communication is possible, the paradox of social interaction, and the need to resolve it by virtual bargaining or a similar mechanism, still arises. Communicative signals—from facial expressions, to gestures, to complex symbolic language—are invariably highly ambiguous (Christiansen & Chater, 2022) and require complex pragmatic processes to determine a specific meaning (Clark, 1996; Grice, 1975; Levinson, 2000; Sperber & Wilson, 1986). Communication will only succeed, of course, where the speaker and hearer alight on the *same* meaning. Thus, the pragmatic inference involved in interpreting a communicative signal is a paradigm example of a coordination problem. Suppose the speaker points vaguely and asks, “Can you pass me that one?” The speaker has to work out (among other things) what the hearer thinks “that one” is; and the hearer has to work out what the speaker thinks “that one” is. This problem of mutual prediction cannot be resolved by any amount of recursive mentalizing (Clark, 1996).

Although further communication may help resolve uncertainty (Healey et al., 2018), any such communication will itself be ambiguous and need further interpretation. Virtual bargaining provides a possible route for breaking out of this regress: in the light of their common ground, both parties ask, “Given our objectives, what would be the most efficient way to map possible signals to meanings?” If they can jointly infer a signal-meaning mapping in the present communicative context, given their common ground, then this mapping can be applied by both parties to interpret whatever signal happens to be sent. For example, if two people are doing some DIY together and have a pile of tools on the floor between them, “that one” must be a screwdriver, given their common ground that their current task is fixing a screw to the wall. If the speaker wanted the hammer right next to the screwdriver, they would need to be more specific (“that hammer”). The shorter, simpler phrase is reserved for the most salient interpretation, given their common ground, which follows standard efficiency considerations (e.g., Gibson et al., 2019). This approach is in the spirit of Clark’s (1996) theory of communication and has been recently developed using the virtual bargaining framework and related work (Bundy et al., 2021; Chater & Misyak, 2021; Stacy et al., 2021).

### How Much Virtual Bargaining is Carried Out “in the Moment”?

An important direction for future work is to clarify whether and when virtual bargaining occurs in the moment, or whether it is better considered as a “rational analysis” (e.g., Anderson, 1990; Chater & Oaksford, 1999) for repetitive behavior that is long-established through individual learning or cultural evolution. This issue arises, of course, throughout psychology: for example, in considering instance-based versus algorithmic theories of skill learning (e.g., Logan, 1988), construction vs rule-based views of syntax (e.g., Ziegler et al., 2019), in Gricean-style natural language pragmatics (Goodman & Frank, 2016), or the interpretation of metaphor (Glucksberg, 2003). Such questions are, of course, typically difficult to answer in any domain. We suggest, though, that the flexibility of social interactions, and their responsiveness to very subtle communicative cues or shifts in background knowledge (see, e.g., the astonishing flexibility of the interpretation of newly encountered communicative signals, e.g., Clark, 1996; Galantucci, 2005; Misyak et al., 2016), suggests that a good deal of processing must be improvised in each fresh communicative interaction. Nonetheless, as with skill learning more broadly, it is likely that each new social interaction will be shaped by prior experience of similar interactions, gradually leading to the “entrenchment” of certain patterns of behavior. Thus, as noted above, systems of increasingly rule-like patterns in behavior would be expected to arise both during learning and development within the individual, and within newly formed social groups. At each point, though, social behavior seems highly malleable and adaptable to the demands of the moment, which seems to defy codification and require in-the-moment adjustment by social actors.

## Conclusions

The shared intentionality of social interaction, and the consequent mutual dependence of minds, is ubiquitous and relatively uncontroversial. Yet it appears to have paradoxical consequences for many theories of the psychological foundations of social behavior. In particular, a theoretical circularity seems to arise where *A*’s thoughts and actions depend on *A*’s interpretation of *B*’s thoughts and actions; yet *B*’s thoughts and actions depend on *B*’s interpretation of *A*’s thoughts and actions. Even formulating this apparent problem of circularity leaves one feeling rather befuddled. It seems initially possible that this might be no more than a pseudo-problem, somehow generated by loose argument or use of language; or perhaps a genuine conceptual puzzle, but one with few implications for the psychologist. We suggest the opposite: the mutual prediction that is essential to social interactions generates a paradox that psychological theories ignore at their peril. Thus, theoretical accounts that see social interaction as involving each person predicting the other’s behavior (Tamir & Thornton, 2018), reading their mental states (Gopnik & Wellman, 1992), or forming first-, second-, or higher-order beliefs about others (e.g., Liddle & Nettle, 2006) will inevitably be incomplete. More generally, theories that aim to explain social behavior in terms of reasons are coherent only to the extent that the reasons that they provide are free of paradoxes. We have seen that in the context of reciprocal social interactions avoiding paradox is surprisingly difficult.

In this paper, we have considered simple social interactions in which the paradox arises very starkly, even though the “natural” solution is highly intuitive. Thus, social interaction seems profoundly different from either social perception (interpreting the behavior of others through observation) or social action (one-way influence on the behavior of others). This observation has, we believe, an important implication for the empirical study of social behavior—that by abstracting away from *interaction*, as has been common in social psychology for many decades, psychologists are missing a crucial aspect of social cognition and behavior.

The solution to the paradox of social interaction requires, we have argued, thinking about what “we” should do—a shift from I-thinking to we-thinking. And the virtual bargaining account aims to explain we-thinking without invoking the shadowy notion of a group mind. Instead, we propose that individuals can we-reason successfully by asking: what would we agree, if we can discuss and bargain? Where the result of such simulated virtual bargaining is self-evident to all parties, then this result can be followed directly. Understanding the nature and limits of virtual bargaining, how people decide who is part of the bargain, how the common ground underpinning virtual bargaining is established, and how the potential for successful virtual bargaining affects whether people wish to join or maintain relations, or group membership, remain interesting questions for future research. As we have seen, virtual bargaining provides a possible mechanism for the cumulative creation and entrenchment of linguistic conventions and social norms. Moreover, the normative force of virtual bargaining—that participants in the bargain feel that they and others ought to follow it—suggests interesting connections with moral psychology.

The argument of this paper has implications for how computational models of nonsocial behavior in the cognitive and brain sciences may extend to social interaction. Computational theories of human cognition, rooted in cognitive science, machine learning, artificial intelligence, and neuroscience, have become increasingly sophisticated and successful. Yet, we argue, these models are, in a specific and important sense, asocial. Specifically, the mutual prediction in social interaction—the fact that each participant is attempting to predict, respond to, and accommodate the thoughts and actions of the other—lies outside the scope of such models. Indeed, the attempt to apply standard models of human cognition to mutual social interaction leads, as we have seen, to paradoxical results.

According to the argument of this paper, the process of virtual bargaining is fundamental to the human ability to engage in complex social interactions. Yet, we are often oblivious to this process of bargaining in part, we suggest, because the highly sophisticated social reasoning underpinning our interactions is so familiar and natural to us. Just as we have the illusion of “direct” contact with the visual environment, unaware of the spectacularly complex calculations carried out by our visual brains (e.g., Ullman, 1980), so the world of social interaction seems “transparent” to us, even though rich and subtle background calculations are in play.

## Figures and Tables

**Table 1 tbl1:** Social Perception, Influence, Transmission, and Interaction in Studies in Social, Developmental, and Comparative Psychology

Area of psychology	Social perception: Understanding the behavior of others	Social influence: Shaping the thoughts and behavior of others	Social transmission: The propagation of thoughts and behavior	Social interaction: Two-way interplay of agent’s behavior
Social psychology	Attribution theory (Weiner, 2018) Interpreting emotions from faces (Todorov et al., 2015) Implicit processes in evaluating others (Greenwald & Lai, 2020)	Obedience (Grzyb et al., 2018) Conformity (Schultz et al., 2007) Mechanisms of persuasion (Cialdini, 2001) Bystander effects (Fischer et al., 2011)	Automatic imitation (Cracco et al., 2018) Emotional contagion (Hatfield et al., 1993) Transmission of beliefs and stories (Heath et al., 2001)	Role-play experiments (Haney et al., 1973) Impact of shared attention on cognition, emotion and affiliation (Haj-Mohamadi et al., 2018)
Social and cognitive development	“Theory of mind” tasks (Wimmer & Perner, 1983) Inferring goals (Gergely et al., 1995; Hamlin et al., 2007)	Socialization of moral norms (Grusec & Goodnow, 1994) Children enforcing social norms on others (Schmidt et al., 2012)	Imitation of facial expressions in neonates (Meltzoff & Moore, 1977; Oostenbroek et al., 2016) Imitating aggressive behavior (Bandura et al., 1961) Rational imitation in infants (Gergely et al., 2002)	Early shared intentionality (Tomasello & Carpenter, 2007) Helping behavior in infants (Warneken & Tomasello, 2007)
Comparative cognition	Chimps infer food location from gaze (Hare et al., 2001) Scrub-jays food-caching depends on what others have observed (Emery & Clayton, 2001)	Machiavellian intelligence: Chimps apparently attempt to mislead others on location of food (Byrne & Whiten, 1989) Animal signaling as manipulation (Dawkins & Krebs, 1978) Collection action in animal groups (Couzin et al., 2005)	Imitation and emulation (Whiten et al., 2009) Cultural learning (Heyes & Galef, 1996) Possible “teaching” between animals (Hoppitt et al., 2008)	Joint attention (Povinelli & Eddy, 1996) Joint action (Bullinger et al., 2011) Cooperative hunting (Boesch, 1994)
*Note*. While interaction seems central to social behavior, many fields of research focus on one-directional relationships between people: how people interpret, influence, or transmit information or behavior to each other. These phenomena can be studied without facing the problem of mutual interdependence that generates the paradox of social interaction: that in social interaction each person is trying to second-guess the thought and behavior of the other.				

**Table 2 tbl2:** Individualistic Approaches to the Problem of Reasoning About Social Interaction in Psychology, Philosophy, and Economics

Type of theory	Outline	Challenge from mutually interdependent interaction	Illustrative references
Theory-theory	Each person formulates a “theory” of the other’s beliefs and desires	Circularity: *A*’s theory of *B* includes *B*’s theory of *A*	Gopnik and Wellman (1992) and Wellman (1992)
Simulation theories	Each person uses their own mind to simulate another’s	Circularity: *A* simulates *B* who is simulating *A*	Goldman (2006) and Gordon (1986)
Prediction-based theories	Each person is a “prediction machine” who best-responds to what she predicts the other will do	Circularity: *A*’s choice depends on its prediction of *B*’s choice; *B*’s choice depends on its prediction of *A*’s choice	Tamir and Thornton (2018)
Truncated recursive theories	Cognitive hierarchy theory; k-level reasoning; higher-order intentionality	Depends on heuristics concerning the “0th” recursive level	Camerer et al. (2004) and Stahl and Wilson (1994)
Bayesian models of mutual prediction	Softmax choice rule and iterate to find “fixed points”	Problem of choosing between multiple equilibria	Shafto et al. (2014)
Rational speech act theory	Iterate to find “fixed points”	Problem of choosing between multiple equilibria	Frank and Goodman (2012)
Nash equilibrium	Each person best-responds to the other’s strategy	Problem of choosing between multiple equilibria	Nash (1950)
Rationalizability	Each person optimizes in light of their own beliefs. Solving for “fixed points”	Problem of choosing between multiple choice vectors	Bernheim (1984) and Pearce (1984)

## Footnotes

1 We would like to thank Garriy Shteynberg, two anonymous reviewers, and the editor for insightful feedback on previous drafts, which has greatly strengthened the paper.

2 Shared intentionality is the term preferred by Tomasello (e.g., Tomasello & Carpenter, 2007); in philosophy, the term collective intentionality is also widely used, though analyzed in a variety of importantly distinct ways (e.g., Bratman, 1993; Gilbert, 1989, 2009; Searle, 1990; Tuomela & Miller, 1988). In economics, related research has been developed under the heading of team reasoning (e.g., Bacharach et al., 2006). Yet further terms, including we-reasoning and we-thinking, are also widely used (Akerlof, 2016; Hakli et al., 2010). Here, we use shared intentionality to cover all these lines of research. Schmid (2012) notes that related ideas about collective thought can be traced to figures as diverse as Habermas (1987), Sellars (1968), Collingwood (1947), and German phenomenologists in the early 20th century. We will consider below how the specific account outlined, based on virtual bargaining, relates to some of these ideas.

3 There are notable exceptions, including Colman (2003), Colman et al. (2008), De Freitas et al. (2019), and Shteynberg et al. (2020).

4 Moreover, social coordination may often arise through the operation of shared cognitive and perceptual mechanisms, which align thought and behavior between people. Such mechanistic accounts, as in influential models of dialogue in psycholinguistics (e.g., Pickering & Garrod, 2004), lie outside the scope of reason-based explanation entirely.

5 We stress that there is nothing inherently paradoxical in recursion per se, which is, after all, a familiar and central in computer science, cognitive science, and mathematics (Hofstadter, 1979). The problem is how such reasoning can resolve the problem of coordination between people who are operating independently. See, for example, Bacharach et al.’s (2006) discussion of the so-called “hi-lo” game, where there are multiple equilibria and the need for some rationale by which people can coordinate on the same one.

6 The analysis here draws heavily on Clark (1996).

7 Common ground might arise from publicly available information, shared culture, a common perceptual environment, collective attention (Shteynberg et al., 2020), or other sources (e.g., Clark, 1996). In economics and philosophy, the more usual term is common knowledge, and here we use these terms interchangeably, following, for example, Thomas et al. (2014). The notion can be spelled out formally, either in set theory (e.g., Aumann, 1976) or epistemic logic (e.g., Harman, 1977; Schiffer, 1972). There is a debate concerning whether Lewis’s (1969) important early formulation is a distinct approach (Cubitt & Sugden, 2003; Vanderschraaf, 1998). Our discussion here does not depend on these fine details.

8 Some authors have argued that experimentally studying social and linguistic *interaction* is of crucial importance. They have not necessarily endorsed the reason-based style of explanation advocated here (Pickering & Garrod, 2004, 2021; Schilbach et al., 2013).

9 We leave open the possibility that social perception, influence, and transmission may sometimes involve some degree of mutual interaction—indeed, this will be the norm in communicative contexts such as our “book donating” example above. Our point is simply that they need not do so.

10 The problem is, in more technical terms, that developing a theory of Level-0 reasoning (which is presumed to be the starting point for higher level reasoning) is in these cases just as difficult as solving the original problem of deciding what the players will do. In practice, Level-0 reasoning typically, though not always, corresponds to a uniform probability distribution over the set of all strategies available to a player.

11 Where levels of mind-reading are evaluated directly, recursive depth is assumed to be very limited. For example, behavioral games, such as the “beauty contest” game (Nagel, 1995), are used to measure recursive depth in competitive interactions. They typically suggest that people may best-respond to the other (i.e., first-order reasoning), or best-respond to a first-order reasoner, but that higher-order reasoning is rare. As a reviewer has pointed out, obtaining predictions from level-k reasoning is complicated further by the fact that depth of reasoning differs between people, and, indeed, is not consistent across games (Georganas et al., 2015).

12 A reviewer has pointed out that the sociologist Max Weber influentially raised skepticism about ascribing beliefs, intentions, or choices to groups or organizations, unless these can be recast in terms of the beliefs, intentions, or choices of the individuals of which these organizations are composed—on the grounds that only individual people have a subjective understanding (*Verstehen*) of their actions. How ascriptions of joint mental states can be ascribed to a group based on the mental states of its members has been an important focus in psychology, philosophy, and economics, and one to which we return below.

13 Indeed, if one party uses private knowledge not available to the other person to choose their interpretation, this will likely disrupt coordination, because the other cannot “follow” the same line of reasoning.

14 The viewpoint requires a “rich” notion of joint action, which we endorse; but some theorists propose more minimalist accounts (e.g., Vesper et al., 2010).

15 This viewpoint is closely linked with important recent computational work in a Bayesian framework on a somewhat different, and in some ways more challenging problem (Kleiman-Weiner et al., 2016; Wang et al., 2021). In this work, the agents do not have the “payoffs” of possible combinations of actions in common ground, but must learn them from experience; and they must also learn the “utilities” of others, and whether the others are collaborating for the benefit of the team, or are maximizing their own rewards (Kleiman-Weiner et al., 2016). Recently, Wang et al. (2021) have proposed an interesting algorithm, Bayesian delegation, in which each agent attempts to infer the tasks of the entire set of agents using Bayesian inference from observing their actions. The payoffs to each agent depend on the interaction of different agents on a task (e.g., collaborating on some subtasks, distributing other subtasks between themselves). Here, the focus is on learning about, and hence coordinating appropriately, with other agents, by observing their actions over long periods of interaction. By contrast, the approach that we develop below, virtual bargaining, focuses on the complementary problem of how agents can instantaneously find a common plan, by reaching an agreement through a process of hypothetical bargaining.

16 We thank an anonymous reviewer for raising this point.

17 Some theorists suggest that such a reduction may not be possible (e.g., Searle, 1990)—that we-thinking needs to be taken as primitive, and not reduced to the mental states of individuals. We shall see shortly that virtual bargaining can be viewed as a particular way of reconciling a distinct “plural subject” (Gilbert, 1989) as being both distinct from, but compatible with, the minds of individual participants (Bratman, 1992, 1993).

18 A reviewer has pointed out that deadlocks can still occur, even when communication is available, such as when two people, not sure who is next in the checkout line, end up politely trading “You go first!” “No, you go!” “No, really, you go ahead” “No, I’m sure you were here before me” sometimes to the point of mutual irritation.

19 A fruitful line of future research is to consider principles of counterfactual or hypothetical thinking observed in other domains, whether hypothetical reasoning about the physical world, or about individual behavior (Byrne, 2005; Roese, 1997). For example, we might expect that bargains that involve easily imagined changes from the status quo will be preferred (Kahneman & Miller, 1986); that people will consider only a very small number of possible bargains (Byrne, 2005; Johnson-Laird, 1983); and that they will be particularly likely to alight on salient or focal bargaining solutions (Schelling, 1960). Perhaps especially intriguing is the finding that social power may shape how readily people imagine that they might act differently (Scholl & Sassenberg, 2014). It is interesting to wonder whether this effect will carry over to tasks involving joint planning through virtual bargaining.

20 Note that Nash bargaining is not connected to the Nash equilibrium mentioned above.

21 Other theories of bargaining could also be used, for example, Kalai–Smorodinsky bargaining, which focuses on a fairness criterion embodied in equalizing the ratio of utility gains (Kalai & Smorodinsky, 1975).

22 Virtual bargaining may help reconcile a dispute in the philosophy of shared intentions. According to Bratman (e.g., Bratman, 1992, 1993), a joint commitment or shared intention requires each member of a group to be personally committing to carrying out their part in a joint plan. According to Gilbert (1987, 2006), it requires instead the group to be committed “as a body,” which cannot be reduced to commitments of individual members. The plot of a Chesterton (1908) novel illustrates how these views appear to diverge. A group of seven putative anarchists plot to overthrow the state, not realizing that none are genuine anarchists (indeed, six are undercover detectives). Gilbert’s account allows that the group intended, as a body, to overthrow the state. Bratman’s account denies this because its individual members do not have this intention. The virtual bargaining account helps capture both perspectives. Given their common ground (crucially not including each individual’s private knowledge of being a detective), each putative anarchist believes that the virtual bargain is to individually and collectively overthrow the state and behaves in accordance with the bargain. Moreover, as Gilbert notes, each member of the group will anticipate, and probably receive, sanction from the others if they fail to “play their part.” Thus, it is the existence of this virtual bargain that makes the group committed to the plot “as a body.” But each individual has private knowledge (that they are detectives) which at some point will cause them to betray the plot. Thus, in line with Bratman’s account, the plot is in a sense entirely illusory, because no individual will ultimately carry it through. Thus, Gilbert’s and Bratman’s perspectives appear compatible rather than contradictory.

23 This observation has been called the “fractal” character of everyday reasoning (Oaksford & Chater, 1998), as each part of the justification for, or explanation of, a belief is as complex as the belief itself. This line of thinking has varied intellectual roots: to coherentist, rather than foundationalist, theories in epistemology (Olsson, 2021); to the origin of the frame problem in artificial intelligence (Pylyshyn, 1987); and to the defeasible nature of human reasoning (Oaksford & Chater, 2007).

24 Although this may sometimes be a useful theoretical approximation (Hawkins et al., 2019).

25 It need not be in common ground to nonparticipants that they are not included—indeed, nonparticipants to social interactions need not even be aware that the interaction is occurring.

26 The Centipede game was originated in Rosenthal (1981). The version we describe here is Rosenthal’s original version, but many variations have since been developed and explored experimentally.

27 The picture is different where the PD is repeated indefinitely; then, the so-called “folk theorem” of game theory applies, which states that (typically) a wide variety of outcomes is rationally justifiable (e.g., Gintis, 2000)—but the players then face the coordination problem of simultaneously alighting on the same one, which has been the primary topic here. Thus, the need for virtual bargaining, or some similar mechanism, recurs in a rational account of infinitely repeated PDs. With one-shot PDs, virtual bargaining does not necessarily generate a cooperative outcome. By contrast, with the finitely repeated PD, a virtual bargain to cooperate throughout is credible, because even if one agent follows the agreement and the other best-responds, this implies that defection will only occur on the last turn, so that both agents can accrue significant benefits for prior cooperation.

28 In evolutionary game theory (Axelrod & Hamilton, 1981), algorithmic strategies such as tit-for-tat are primitive behavioral patterns that are selected for over repeated interactions between agents. Thus, the algorithms that predominate are those that have met with greatest success in the past. By contrast, in the virtual bargaining approach, strategies (which may include tit-for-tat) arise through a process of forward-looking choice, in view of their likely success in the current interaction.
